# Is unrestricted weight bearing immediately after fixation of rotationally unstable pelvic fractures safe?

**DOI:** 10.1186/s12891-022-05299-5

**Published:** 2022-04-11

**Authors:** William E. C. Poole, David W. Neilly, Mark S. Rickman

**Affiliations:** 1 Orthopaedic Trauma Department, Royal Adelaide Hospital, Adelaide, Australia; 2 Trauma & Orthopaedics, University of Adelaide, Adelaide, Australia

**Keywords:** Pelvic trauma, Early weight bearing, Pelvic fixation

## Abstract

**Introduction:**

Rotationally unstable pelvic fractures treated with surgical fixation have traditionally been treated with restricted weight bearing on the affected side for 6–8 weeks post operatively.

We have been developing pelvic fixation standards to allow for unrestricted weight bearing immediately post operatively in type B rotationally unstable pelvic fractures.

**Aims:**

To assess for safety and efficacy of allowing unrestrictive weight bearing in this cohort of patients, we have clinically and radiologically monitored outcomes up to two years post operatively.

**Methods:**

Through retrospective review, two cohorts of patients with Tile Type B pelvic fractures were identified that were treated at the Royal Adelaide Hospital, South Australia.

Patient demographics, injury classification, surgical fixation and weight bearing status post operatively was recorded.

One cohort of patients was allowed to fully weight bear post operatively, whilst the other was treated with 6 weeks of restricted post op weight bearing.

At clinical follow up, post-operative x-rays were assessed for loss of reduction, screw or plate breakage and reoperation.

**Results:**

Between January 2018 and January 2021, 53 patients with rotationally unstable pelvic fractures that underwent surgical fixation were included in this study.

One group of patents were allowed to immediately weight bear as tolerated (WBAT) post operatively (*n* = 28) and the other with restricted weightbearing (RWB) (*n* = 25).

There was 1 re operation for failure of fixation in each group.

Metalwork breakage was more common in the WBAT group than in the RWB group and this was seen only in APC fractures. This increase in metalwork failure was not associated with loss of reduction.

**Conclusions:**

With surgical fixation, Tile type B rotationally unstable pelvic fractures can be allowed immediate weight bearing post operatively.

We found this to be safe and effective, employing surgical strategies to address both anterior and posterior injuries to allow immediate unrestricted weight bearing.

Broken metalwork was more commonly seen in the WBAT group but this was not associated with loss of reduction or reoperation.

## Introduction

Patients with rotationally unstable pelvic ring injuries benefit from operative fixation to restore bony anatomy and enable early rehabilitation and recovery [1]. Successful management of pelvic fractures requires the interpretation of clinical and radiographic information to assess fracture stability. Pelvic fractures are commonly classified using the Young and Burgess [2] and Tile [3] systems. Young classified pelvic ring injuries according to the mechanism of injury, whereas Tile described injuries related to stability both rotationally and vertically. In 2018 the AO/OTA fracture classification [4] was revised and is now commonly used.

Unstable pelvic fractures that require surgical stabilisation are typically composed of anterior and posterior segments. Surgical management of pelvic fractures aims to restore bony anatomy and subsequently maintain this position until bony union or ligamentous stability. Anterior fixation is often achieved with open reduction and fixation using plates and screws, whilst the most common modality of fixation for the posterior injury remains ilio-sacral screws (ISS). Some fracture patterns and associated soft tissue injuries require the usage of external fixators for stabilising the anterior pelvic injury in conjunction with internal stabilisation posteriorly. Posterior fractures with significant comminution or displacement that cannot be reduced closed, may require open reduction prior to internal fixation.

Debate remains amongst pelvic surgeons around the best method of treating rotationally unstable but vertically stable pelvic fractures. The findings from a recent survey of British and Irish pelvic consultants predominantly favoured an anterior plate and one ISS [5] with the majority of these surgeons not permitting full weight bearing on the affected side until weeks 8–12 post operatively.

Since 2018, we have been developing standards in our level 1 trauma centre with pelvic fracture care allowing for surgical fixation with immediate unrestricted weight bearing in rotationally unstable (type B) pelvic fractures. Little evidence exists pertaining to weight bearing following pelvic fracture surgery as summarised in a recent systematic review [6]. Expert opinion but without a strong evidence base has traditionally limited post-operative weight bearing following pelvic surgical fixation. Rotationally unstable type B fractures treated with anterior and posterior fixation traditionally have been treated with restricted partial weight bearing for 6–8 weeks post operatively.

Early weight bearing following pelvic fracture surgery in theory would afford the potential benefits of maintaining bone stock, muscle mass and joint range of movement whilst simultaneously improving the rehabilitation goals of the patient. There are added benefits of reducing the risk of venous thromboembolism and potential positive psychological benefits following trauma which would be beneficial when caring for these complex patients. Early weight bearing also can increase return to work and reduce the financial burden of trauma [7].

Immediate unrestricted weight bearing should only be employed if it were found to be safe and not to result in loss of reduction and subsequent reoperation. Following this evolution in our practice we have carefully reviewed all pelvic fracture patients treated between January 2018 and January 2021, to assess the safety of this surgical philosophy.

## Methods

### No ethical approval was required for this retrospective, single centre study

A retrospective review of all patients with pelvic fractures from Jan 2018 to Jan 2021 that received surgical fixation at the Royal Adelaide Hospital, South Australia was performed. Data including patient demographics, date, time to surgery, and mechanism of injury was collected. All fractures were classified based on both the mechanism of injury and radiographic appearances [2–4].

Patients were split into two groups based on their post operative weight bearing status as documented in the notes; immediate weight bear as tolerated (WBAT) and restricted weight bearing (RWB). Weight bearing status was decided by the treating consultant surgeon.

For this analysis, Vertical shear patterns were excluded from the data set, as were vertically and rotationally unstable patterns and patients with combined acetabular and pelvic fractures.

The method and type of fixation both anterior and posterior was recorded alongside whether the patient had suffered multiple injuries, with those other injuries also noted.

Weight bearing status following pelvic fixation was scrutinised, both in the immediate post operative period and during clinic follow up. Those with weight bearing limited by other injuries were noted. The polytrauma patients with RWB from other injuries were included in the pelvic RWB groups.

All patients were treated by one of three fellowship trained pelvic consultant surgeons; most of these cases were performed by the senior author (MR). The post-operative weight bearing status was decided by the treating surgeon with increasing confidence of immediate WBAT as the study period progressed.

Length of follow up was documented, alongside any loss of reduction on radiographs, or re-operation. Standard follow up was at 2, 6 weeks and 12 weeks clinically and with radiographs, and then extended depending on the injuries and progress of the patient. Patients with follow up of less than 3 months were excluded. Evidence of radiographically confirmed venous thrombo-embolism – deep vein thrombosis (DVT) or pulmonary embolus (PE) on venous duplex ultrasound or CT pulmonary angiogram was also recorded.

## Results

The patient demographics are presented in Table 1.

**Table 1:** Demographics

Total number of fractures treated surgically	78
Total of type B unstable pelvic fractures	53
M:F	36:17
Age average	45.7 years
Age range	19–92

The fracture characteristics of the injuries treated are delineated in Table 2.

**Table 2:** Fracture characteristics

Total type B fractures	53
WBAT:RWB	28:25
Follow Up	
WBAT	3-30 months Avr 9.3 months
RWB	3-32 months Avr 10.5 months
Fracture types	
AnteroPosterior Compression (APC)	Lateral Compression (LC)
APC 2 / type B n = 29	LC 1/2 type B n = 24
AO 61 B2.3 = 23, 61 B3.3 = 6	AO 61 B1.1 = 11, AO 61 B2.2 = 13
Male 28 (97%): Female 1 (3%)	Male 8 (33%): Female 16 (67%)
Age avr. 46.9 years Range 22–79	Age avr. 44.4 years Range 19–92
WBAT 13	WBAT 15
RWB 16	RWB 9

All but one of our APC fractures were in male patients (28/29) with the majority being as a result of being in a car or motorcycle collision (18/29).

Comparatively the majority of our LC fracture patients were female (16/24) and their injuries were more commonly sustained after falls (10/24).

10/28 of the WBAT group were polytrauma patients whereas a slightly higher proportion 13/25 were in the RWB.

On average patients had to wait 3.6 days for surgery (range 0-13 days). Of the 53 patients 4 waited for over 10 days for fixation but these all had immediate trial of mobilisation and after Xray at 7 days were found to have dynamic instability and limited rehabilitation progress.

As our experience in immediate WBAT has grown we have increased the number of patients that are permitted this post operative prescription, particularly in the APC group, this is illustrated in Table 3.

**Table 3:** Patient percentage weight bearing year by year

**Year**	**ALL WBAT:RWB**	**% WBAT**	**APC WBAT:RWB**	**%WBAT**	**LC WBAT:RWB**	**%WBAT**
2018	10:12	45%	4:9	31%	6:3	66%
2019	6:6	50%	3:4	43%	3:2	60%
2020	12:7	63%	6:3	66%	6:4	60%
total	53		29		24	
**Year**	**Total WBAT %**					
2018	45%					
2019	50%					
2020	63%					

In Tables 4 and 5 we describe how we surgically managed the APC and LC fractures in each group.

**Table 4:** APC fracture fixations

APC2/type B *n* = 29	
WBAT *n* = 13 (AO B2.3 = 9, AO B3.3 = 4) (2 bladder injuries, no polytrauma restricting WB, polytrauma *n* = 5) Polytrauma 38%	RWB *n* = 16 (AOB2.3 = 14, AO B3.3 = 2) (2 abdominal wall injuries – 1 rectus avulsion, 1 adductor avulsion) polytrauma with injuries restricting wb *n* = 4, polytrauma =8) Polytrauma 50%
Anterior open reduction internal fixation (ORIF) only: 1	Anterior ORIF only: 2
Anterior ORIF plus percutaneous posterior 12	Anterior ORIF plus percutaneous posterior 11
	ORIF front and back: 1
	Ex-fix plus posterior fixation: 2

**Table 5:** LC fracture fixations

LC2/type B – n = 24	
WBAT *n* = 15 AO B1.1 = 6 AO B2.2 = 9 (no PT restricting WB, polytrauma n = 5) Polytrauma 33%	RWB *n* = 9 AO B1.1 = 5 AO B2.2 = 4 (PT restricting WB n = 2, polytrauma n = 5) Polytrauma 55%
Anterior ORIF + posterior perc (multiple): 2	Posterior ORIF only: 1
ORIF Anterior + posterior: 1	ORIF Anterior + posterior: 1
Anterior Ex-fix plus posterior fixation: 1	Anterior Ex-fix plus posterior fixation: 3
Anterior + Posterior percutaneous: 11	Anterior + Posterior percutaneous: 4

All posterior percutaneous fixation had 2 or more ISS in the LC fracture fixations allowed to WBAT

### Reoperations and loss of reduction

There were 2/53 cases of acute reoperations (3.7%) (excluding all the planned removal of external fixators used as definitive management at 6–12 weeks). 1/28 in WBAT and 1/25 in RWB.

The first reoperation was in the APC WBAT cohort with an ISS backing out at 3 months that required revision and another screw implanted – this did not result in any loss of reduction.

The second reoperation was in the RWB group where an APC fracture in a patient with sacral dysmorphism had early removal of one of the two ISS at 3 months (S2) after complaining of radicular-like pain symptoms. One S1 screw and a 6H anterior symphyseal plate remained in situ. During follow up review at 6 months the patient experienced anterior and posterior pelvic pain, symptomatic diastasis and failure of metalwork. This patient went on to have 90–90 anterior double plating with iliac crest bone grafting and further reimplantation of a second ISS and then healed uneventfully by 6 months, pain free clinically and fused on CT.

In total there were 4 patients who suffered loss of reduction and mal union. We defined this as recurrent diastasis or loss of fixation with displacement from original position. 1 patient in the WBAT group with a more complex LC2 suffered broken metalwork anteriorly with loss of reduction of the ramus fixation. The posterior plate and screws maintained reduction and fixation. They were asymptomatic from the loss of anterior fixation, remained independently mobile and did not require revision surgery.

There were 3 patients in the RWB group who lost reduction, one of these in an APC fracture required re operation as described above.

The other 2, also APC injuries, were followed up, remained asymptomatic and were treated without revision.

#### Broken metalwork without loss of reduction

We observed broken metalwork in 6/53 cases.

None of the cases with LC type fractures experienced broken metalwork.

In the APC group 6/29 experienced metalwork breakage.

Of the APC patients allowed to immediately WBAT 4/13 were found to have broken screws/plates but without loss of reduction or migration.

2/16 in the APC group with RWB had broken metalwork without loss of reduction.

#### VTE

VTE was seen in patients with polytrauma and pelvic fractures.

One pre operative deep vein thrombosis (DVT) was found in a patient with bilateral open pilon fractures – they had bilateral below knee DVTs and were in the RWB group post pelvic fixation.

Two post-op pulmonary embolus (PEs) in RWB group (both had post op prophylactic LMWH postop and on discharge). Both were again polytraumatised patients, one with a 3C open ankle fracture (1 month post op PE) and one with upper limb injuries (3 months post op PE).

No VTE was seen in WBAT group.

All patients received between 2 and 6 weeks of LMWH post op prescribed on surgeon preference. 37/53 (70%) received a prescription for 6 weeks LMWH.

## Discussion

We present a series of patients with rotationally unstable type B pelvic fractures that were allowed immediate full weight bearing after surgical fixation. As this study progressed, we carefully analysed radiological outcomes to ascertain if patients experienced a loss of reduction or failure of fixation after allowing early weight bearing, and compared outcomes to those that had restricted weight bearing.

### Reoperations and metalwork breakage

We reviewed comparative matched cohorts of patients in regards to age, sex and fracture classification with similar follow up periods. Overall our complication rate of loss of reduction and reoperation is low for both groups of patients. We present our data on patients allowed to WBAT immediately post op to allow surgeons to challenge the dogma of restricting all patients to prolonged periods of restricted weight bearing following surgical fixation.

In all of our patients with LC and APC type B injuries allowed to WBAT immediately post op we experienced only one early complication, with one ISS migration at three months requiring removal and re implantation of another screw. We noted a higher proportion of patients with metal work breakage (either screw or plate) compared with our RWB cohort but these patients did not experience loss of reduction nor were they symptomatic from broken metalware. Interestingly metalwork failure was only observed in the APC group. Hardware breakage is common following pubic symphysis fixation and is reported in the literature and not thought to be clinically important [8]. There is physiological movement at the fibrocartilaginous symphysis after the healing process has completed, and on repeated loading the stress exceeds the fatigue strength of the construct resulting in failure.

Evidence to support addressing both the posterior and anterior ring injury in APC fractures is provided by Putnis. et al. [9] in a series of 49 patients with mostly AP compression type injuries with symphyseal diastasis. A higher revision rate and loss of anterior fixation if no posterior fixation method was utilised. Whilst 15/49 (30%) had evidence of movement/metalwork failure, only 4/49 required revision surgery for recurrent diastasis and all of these had no posterior fixation.

In the patient experiencing screw migration, following further history taking they admitted to strenuously exercising repeatedly on a static exercise bike from the immediate post op period. Whilst it is desirable to allow our patients to exercise and maintain physical condition when recovering from pelvic trauma and surgery, we perhaps had not considered that degree of early stress to our fixation (6-hole symphyseal plate with 1 long S1 screw and 1 long S2 screw).

### Surgical Strategies to consider allowing immediate weight bearing

The method and rigidity of posterior fixation of pelvic injuries remains a challenging decision for the treating pelvic surgeon. The posterior ring contributes nearly 60% to pelvic stability [10]. Posterior fixation with percutaneous ISS has become increasingly common and through published techniques has been found to be safe with the use of either 2D or 3D fluoroscopy with reviews of these techniques available [11–13].

More than one ISS to fix posterior injuries amenable to percutaneous fixation is preferred to enable increased stability of fixation [14] and allow immediate weight bearing. The published literature on this is limited to biomechanical studies but in our series all our lateral compression fracture patients that were allowed to WBAT had 2 or more screws to fix the posterior injury. When it was safe to do so, longer screws (beyond the midline) were used. Longer screws afford greater biomechanical stability as shown in a finite element model of Tile C pelvic ring injuries [15]. ISS diameter is another unresolved issue within the published literature. We have used 6.5 mm screws for all of our ISS.

Transiliac screws are useful in certain fracture patterns and in patients with poor bone stock [16]. These screws can enable the treating surgeon to have increasing confidence when allowing early weight bearing particularly in osteoporotic patients. Scrutinising the posterior osseous fixation pathways on CT for all patients to ensure anatomy that allows for it is paramount to minimise risk of complications with awareness and planning for sacral dysmorphism is imperative. Intraoperative care to screen the contralateral sacroiliac joint mainly in APC patterns and appreciate the injury so as not miss an occult B3 type with bilateral posterior instability is important. Importantly for this technique traversing non-injured SI joints with transiliac screws does not seem to be associated with morbidity [17]. For our ISS we employ a combination of partially threaded 6.5 mm screws as lag screws or fully threaded 6.5 mm screws as a positional screws.

We did not use locking plates or large fragment screws in any of the anterior plate fixations and there remains no consensus in the literature regarding these with surgeons developing their own preference and practice.

Very comminuted anterior injuries that require significant soft tissue dissection and potentially sub optimal fixation with open reduction and internal fixation can be treated with anterior external fixation. Some centres would advocate the usage of the in-fix system for these injuries [18, 19]. There may be a role for this technique in obese patients however we have found supra acetabular anterior ex-fixs to be durable and patients with type B pelvic fractures can still be allowed to WBAT. The use of anterior external fixation when the pelvic fracture is found in conjunction with a bladder injury also remains a reliable choice to reduce the risk of infection.

Percutaneous fixation of pelvic fractures is an appealing option for patients and surgeons alike – less surgical exposure and therefore risk to the patient and hypothesised quicker recovery time. Some fracture patterns are more amenable to percutaneous fixation and those with more displacement may require mini open approaches for reduction. In our series the rami fractures treated with anterior column screws were all via retrograde insertion (from the symphysis towards the gluteus medius pillar). Utilising intra operative fluoroscopy with AP, inlet and obturator oblique outlet views we have found these screws to be safe to implant, although in some patients with a smaller osseous fixation corridor bypassing the hip safely is not always possible with a 6.5 mm screw. In these cases a 4.5 mm large fragment screw or 3.5 mm small fragment screw provides an alternative albeit less rigid option. None of the fractures in this series needed a 3.5 mm column screw. Anterior fixation of lateral compression (LC) fractures when performed is traditionally with open reduction and plate fixation, but selected LC fractures may be amenable to a percutaneous anterior column screw (ACS) or an ‘LC2 screw’ along the supraacetabular osseous tunnel fixing the iliac crescent fracture [20]. These methods of fixation allow maintenance of reduction and ability to weight bear whilst causing significantly less surgical insult to the patient. Employing percutaneous fixation allows for the pelvic haematoma from the fracture to remain undisturbed and thus the risk of haemorrhage is reduced [21].

A useful and descriptive review article on percutaneous pelvis fixation fluoroscopy is available [22]. 73% of our WBAT patients in the LC group had percutaneous fixation front and back. Elderly patients with fragility LC type B injuries were given a 48 h trial of mobilisation prior to offering surgery – if they were unable to mobilise we have found reliably percutaneous screw fixation in these patients provides significant pain relief and subsequent ability to begin to weight bear. Several centres have published promising results in elderly patients aiming to preserve mobility and independence following these injuries [23] and to reduce pain [24], and we echo these sentiments when treating the elderly pelvic fragility fractures.

### Evidence to support early weight bearing

In Tornetta’s paper of rotationally unstable pelvic fractures managed with surgery, there were 29 patients (1),mostly with APCs (23/29), two thirds of which did not have an associated acetabular fracture (19/29) and were allowed immediate weight bearing. They presented no loss of reduction that required reoperation and high return to work within a year (83%). The majority (76%) were ambulating independently at final follow up and only 1/29 complained of pain affecting normal daily activities. A more recent paper by Marchand [25] compared two large cohorts of patients following pelvic fracture fixation from 2006–2015. In this study, early unrestricted weight bearing was classified as before 8 weeks but with no surgeon allowing immediate weight bearing. The RWB cohort were allowed to WBAT after 8 weeks. From this large series they presented an overall low complication rate and low loss of reduction requiring revision and also found time to weight bearing was not associated with complication. There is very little else in the published literature regarding early weight bearing after fixation of pelvic fractures.

### Limitations

We accept the limitations inherent with this retrospective series of 53 patients with pelvic fractures however feel this review of our practice can enable other pelvic surgeons to consider more progressive rehabilitation protocols for their post-operative rotationally unstable pelvic fracture patients. As our experience and confidence has grown we have been able to surgically treat patients and allow more of them to be WBAT immediately post op (45% in 2018 to 63% of patients in 2020).

Within the classification of APC and LC fracture patterns there is heterogeneity and the treating pelvic surgeon will appreciate the subtle findings on Xray, CT and intra operative fluoroscopy. Pelvic fracture stability is a whole scale, and whilst any classification will have discreet categories (eg APC1 vs 2) in real life there is a spectrum. Thus not all type B injuries are equal, and despite the classifications some are more unstable than others.

There are certain signs of possible increasing vertical instability seen in type B patterns, such as large crescent fractures, sacral comminution, vertebral lumbar transverse process fractures or avulsion fractures of the ischial spine which should give surgeons index of suspicion for increasing instability. These have to be added into the “personality” of the injury when deciding on which patients might be suitable for WBAT. Not to mention the personality of the patient!

The scope of some of these type B injuries ‘rotationally unstable and vertically stable’ means protected weight bearing post operatively may well still be more suitable for these selected patterns and rehabilitation protocols should be made post operatively on a case by case matter.

Some of our patients in this group had multiple injuries, in particular some had lower limb injuries that restricted their weight bearing separate to their pelvic fixation (6/53 patients). We included these into our study in the RWB groups as these patients received a period of post op restrictions similar to conventional pelvic management. Several of these patients from a pelvic point of view would have been allowed to WBAT. Adding these patients to the RWB groups resulted in a higher percentage (13/25–52%) of multiply injured patients in this group. Notably more of the RWB group were treated with external fixation for their anterior pelvic ring injury, this may suggest more severe trauma in these patients.

Allowing our patients to WBAT immediately post operatively does not always mean they will be fully weight bearing from day 1. It does allow the physiotherapists to set full immediate weight bearing as a goal for these patients and tailor realistic and specific rehabilitation. It maybe also be true that patients with RWB status may load the fixation more than our post operative prescription specifies. To allow for WBAT the physiotherapists can educate the patients regarding progressive activities during their early rehab. Further work into how much weight these patients are tolerating immediately post operatively would be of interest and is likely to be of broad spectrum with multiple contributing factors. Evidence to consider when RWB in patients, is the demonstration that pressure on the acetabulum during movements such as sit to stand, which are not typically restricted, far exceed those contact forces seen in normal walking [26] as well as patients permitted only touchdown weight bearing requiring four times the energy for walking compared with the average population [27]. We did not use force plates or scales to measure the amount of weight that patients were putting through their limbs. For future work this would provide useful information to know how much weight patients were able to tolerate in the unrestricted weight bearing group or how weight was restricted in the other group.

Within our two cohorts of patients we did not prospectively collect any patient reported outcomes and note that this is one of the weaknesses of our data. We hypothesise that allowing WBAT will help the patients recovering from their injuries, potentially avoiding longer periods of time in either rehabilitation units or in altered living at home, evidence from rehabilitation units exists to support this [28]. Psychological benefits of early weight bearing are associated with the return to functional status. This is seen particularly in patients recovering from hip fractures [29]. The theoretical benefits of early weight-bearing including maintaining bone, muscle mass and joint range of movement are again all avenues that could be researched.

Some of our patients received only 3 months of follow up – this was only in the cases that were deemed uncomplicated, with patients that had been WBAT without pain for some time and without issue seen on serial radiographs.

We looked for evidence of VTE in both groups both before and after surgery. One pre operative DVT and 2 post operative PEs were seen in the RWB group. All of these patients were polytrauma, with 2/3 having severe lower limb injuries as well as their pelvic fractures. This review is clearly underpowered to find any difference in VTE rate between early and late WB pelvic fracture patients. Whist the post operative VTE prophylaxis treatment remains without consensus and lacking high quality studies in the literature [30], and we present heterogeneity in our post op prescriptions from 2–6 weeks of low molecular weight heparin, early mobility post trauma and surgery is one method of reducing risk.

## Conclusions

Within the limitations of this paper we present a cohort of type B pelvic fracture patients some of whom were allowed immediate unrestricted weight bearing after fixation without an increase in loss of reduction or reoperation. In a topic of pelvic surgery where data is extremely limited, the option for the treating pelvic surgeon and the patient with the type B pelvic fracture to bear weight and speed up their recovery is attractive. Further research in this field with patient reported outcomes, cost analyses with times to discharge and force plates to measure how much weight patients can tolerate immediately after fixation are all interesting avenues.

## Data Availability

The datasets generated and analysed during the current study are available in the OSF.io repository. Persistent web link to the database; https://mfr.osf.io/render?url=https://osf.io/r2gxe/?view_only=None%26direct%26mode=render%26action=download%26mode=render

## Abbreviations

WBAT: Weight bear as toleration
RWB: Restricted weight bear
ISS: Ilio-sacral screw
DVT: Deep vein thrombosis
PE: Pulmonary embolus
APC: Anteroposterior compression
LC: Lateral compression
CT: Computed tomography
ACS: Anterior column screw
